# Broadband and Tunable Light Harvesting in Nanorippled MoS_2_ Ultrathin Films

**DOI:** 10.1021/acsami.0c20387

**Published:** 2021-03-09

**Authors:** Mukul Bhatnagar, Matteo Gardella, Maria Caterina Giordano, Debasree Chowdhury, Carlo Mennucci, Andrea Mazzanti, Giuseppe Della Valle, Christian Martella, Pinakapani Tummala, Alessio Lamperti, Alessandro Molle, Francesco Buatier de Mongeot

**Affiliations:** † Dipartimento di Fisica, Università di Genova, Via Dodecaneso 33, 16146 Genova, Italy; ‡ Dipartimento di Fisica and IFN-CNR, Politecnico di Milano, Piazza Leonardo da Vinci, 32-20133 Milano, Italy; § CNR-IMM Unit of Agrate Brianza, via C. Olivetti 2, Agrate Brianza, I-20864, Italy

**Keywords:** large area 2D semiconductors, MoS_2_ nanosheets, broadband photon harvesting, 2D metasurfaces, flat optics

## Abstract

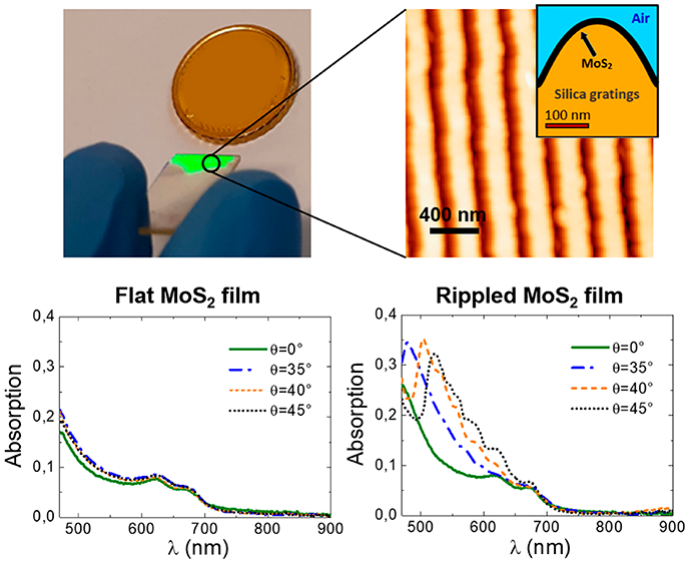

Nanofabrication of flat optic silica gratings conformally layered with two-dimensional (2D) MoS_2_ is demonstrated over large area (cm^2^), achieving a strong amplification of the photon absorption in the active 2D layer. The anisotropic subwavelength silica gratings induce a highly ordered periodic modulation of the MoS_2_ layer, promoting the excitation of Guided Mode Anomalies (GMA) at the interfaces of the 2D layer. We show the capability to achieve a broadband tuning of these lattice modes from the visible (VIS) to the near-infrared (NIR) by simply tailoring the illumination conditions and/or the period of the lattice. Remarkably, we demonstrate the possibility to strongly confine resonant and nonresonant light into the 2D MoS_2_ layers via GMA excitation, leading to a strong absorption enhancement as high as 240% relative to a flat continuous MoS_2_ film. Due to their broadband and tunable photon harvesting capabilities, these large area 2D MoS_2_ metastructures represent an ideal scalable platform for new generation devices in nanophotonics, photo- detection and -conversion, and quantum technologies.

## 1 Introduction

Two-dimensional (2D) transition metal dichalcogenide (TMD) semiconductors have recently emerged as very promising platforms for new-generation atomic devices, due to their unique physical^1−3^ and chemical properties.^4−6^ Novel configurations employing TMDs and their van der Waals heterostructures^7−9^ extending from monolayer to multilayer thickness offer rich prospects in diverse domains like nanophotonics,^10−12^ optoelectronics,^13−15^ quantum technologies,^16,17^ and photocatalysis^9,18^ due to the tunable optoelectronic properties. In particular, the possibility to tailor the electronic band structure of 2D TMDs such as MoS_2_ achieving a transition from indirect to direct bandgap semiconductor in the monolayer form is very promising for applications in atomic scale photonics like photodetection,^10,19^ saturable-,^20,21^ or two-photon absorption,^22,23^ and nonlinear optics.^24−27^ However, exploiting 2D materials as an active layer in nanophotonic devices is hurdled by low photon absorption due to the reduced thickness (monolayer MoS_2_ or WS_2_ absorb around 10% of visible light).^8,28^

Possible solutions to address this critical issue involve novel strategies based either on plasmonic metasurfaces^29−31^ or on flat-optics configurations.^32−34^ The latter, being based upon reshaping the 2D layer in the form of highly ordered subwavelength lattices,^32,33,35^ provides a unique opportunity to control the electronic band structure due to local strain engineering^29,36,37^ and, in parallel, to couple the incoming photons with the active material allowing to tailor the excitation of lattice resonances and/or guided mode anomalies.^38−41^ These narrowband diffraction modes emerging in monodisperse periodic templates induce strong in-plane light scattering and near-field confinement in proximity of the active 2D layer, thus enabling resonant enhancement of the photon absorption.^41−43^

In parallel, the scalable nanofabrication of 2D TMDs is a relevant issue in view of real-world photonic applications since current state-of-the-art devices generally rely on exfoliated 2D flakes, whose area is typically limited in the range of ten square micrometers and shows numerous drawbacks. In particular, the lack of systematic control on the thickness and on the lateral size requires time-consuming morphological characterization steps and complex setup for optical characterization, while device nanofabrication can be uniquely achieved via high resolution nanolithography. To circumvent these issues, new techniques are currently investigated to grow large-area TMDs layers^44,45^ with the further benefit of reshaping the 2D layers via conformal growth on top of self-organized nanopatterned templates.^46−49^ This way, the capability to homogeneously tailor the optical and vibrational response of 2D MoS_2_ layers has been demonstrated over large-area. However, the intrinsic size-dispersion of the nanopattern inhibits the emergence of coherent optical diffraction effects which would strongly promote light confinement in the 2D material.

In this work, we combine laser interference lithography (LIL) with reactive ion etching (RIE) to fabricate well-ordered silica gratings that are scalable to a macroscopic area and are used to drive the nanoscale reshaping of few-layer 2D MoS_2_ grown on top. The subwavelength period of the gratings allows for the emergence of a polarization sensitive guided mode anomaly (GMA) that promotes the enhanced photon absorption in the conformally nanorippled 2D MoS_2_ film which acts as a flat optic element. We demonstrate the capability to tune the resonant wavelength of the GMA in a broadband spectral range from the near-ultraviolet (NUV) to the NIR by changing the illumination angle and the grating period, thereby achieving relative enhancement of photon absorption up to 240% with respect to a comparable MoS_2_ film grown on a flat silica substrate. Additionally, the high amplitude and subwavelength modulations of the hybrid 2D-TMD/SiO_2_ rippled interface induce a broadband biomimetic reduction of reflectivity due to the refractive index grading (moth-eye effect).^50^ The capability to tailor broadband photon harvesting combined with antireflection functionalities over large-area templates highlights the potential of metastructures based on 2D MoS_2_ for applications in nanophotonics, optoelectronics, and quantum technologies.

## 2 Results and Discussion

The nanofabrication of large area flat optic metasurfaces, based on few-layer MoS_2_ homogeneously coating the top of nanopatterned silica substrate, has been achieved by combining LIL and RIE techniques followed by deposition of few-layer 2D MoS_2_. More in detail, we use LIL at two different illumination angles to impress optical fringes with different periodicity on a polymer coated flat silica substrate followed by deposition of aluminum (Al) mask at normal incidence. The Al coated silica template is then processed through RIE, leading to the formation of high amplitude subwavelength gratings on the silica surface with desired period (see the Methods section for experimental details). The use of RIE enables directional etching of the silica surface, allowing us to modify the surface profile in a controlled manner by regulating the exposure time of the sample to the active agents in the etching process. The robustness of LIL and RIE allows us to fabricate controlled subwavelength gratings extending over large area (cm^2^) on transparent silica substrates that are used as templates to grow thin conformal precursor film of MoO_3_ using electron beam evaporation followed by sulphurization, then leading to the formation of a 4–5 layer thick MoS_2_ corresponding to a thickness of 2.8−3.5 nm (see the Methods section for details). Indeed, the periodic modulation of the active 2D layer allows us to control the electronic band structure through local strain engineering, leading to tailored optoelectronic response.^36,37,49,51^

**Figure 1:**
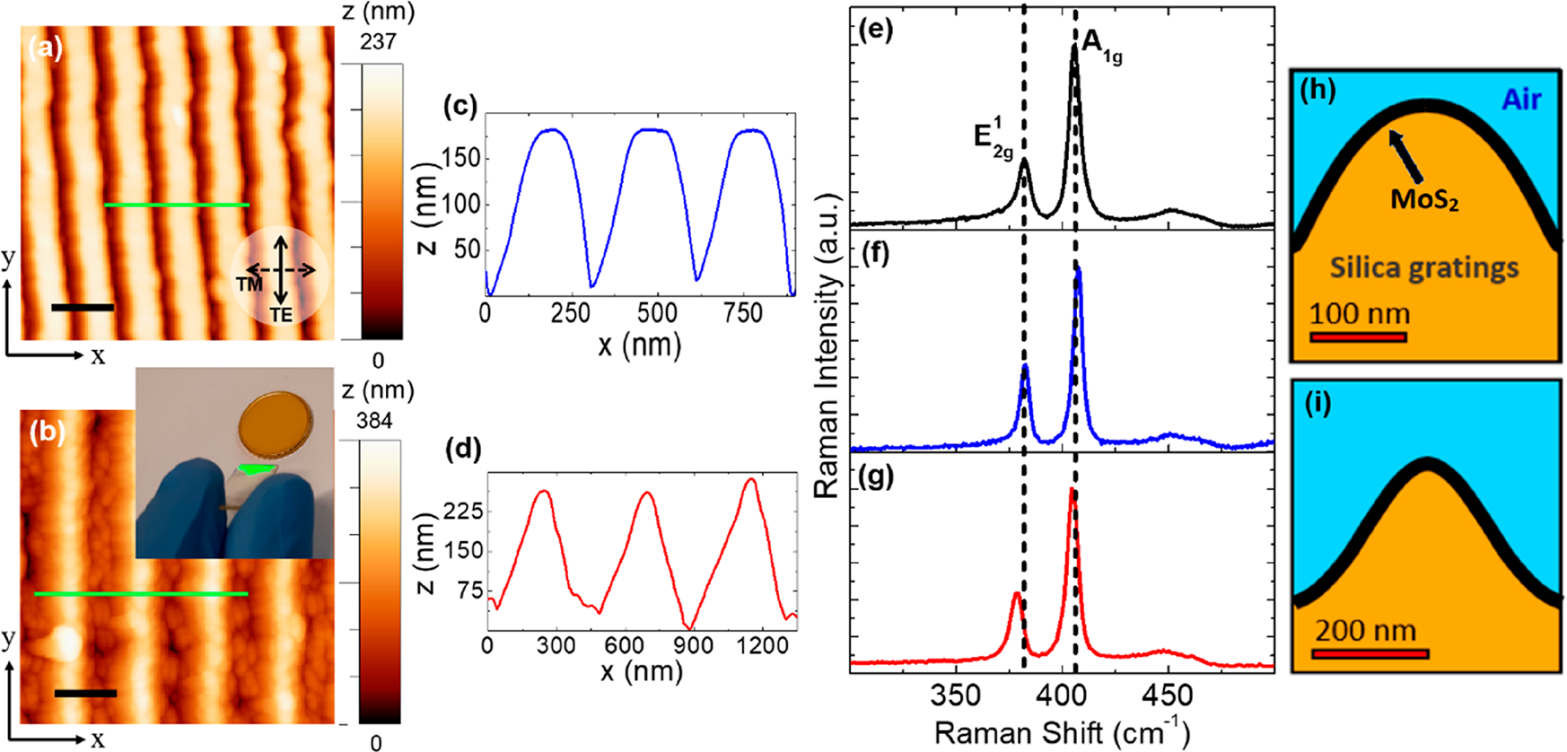
(a,b) AFM images for Samples 1 and 2, respectively. Scale bar reads 400 nm. Inset of panel 1b shows large-scale photograph of Sample 1. (c,d) Line profiles corresponding to the horizontal green line in AFM images of Samples 1 and 2, respectively. (e–g) Raman spectrum acquired for a flat MoS_2_ reference film (black curve) compared to the spectrum from Sample 1 (blue curve) and Sample 2 (red curve). (h,i) Finite Element Method (FEM) geometry used in the simulations for Samples 1 and 2, respectively. Regions with different refractive indices have been marked in the unit cell employed for Sample 1 (the thickness of the MoS_2_ layer is not to scale in the sketch).

The surface morphology of silica gratings decorated by a few layer MoS_2_ film is imaged through atomic force microscope (AFM) as represented in Figure 1a,b (and schematically sketched in Figure 1h,i). The deposition of the flat reference MoS_2_ films of comparable thickness has been previously reported in ref (52). From the statistical analysis of the AFM images, the grating period (*D*) reads (290 ± 15) nm for Sample 1 (Figure 1a) and (450 ± 15) nm for Sample 2 (Figure 1b). The controlled exposure of the silica surface to active agents in RIE allows us to easily modify the surface morphology and the amplitude of the gratings as shown in line profiles in Figure 1c,d, corresponding to the AFM image shown in Figure 1a,b. A broad ridge is observed for Sample 1, whereas a sharp conical profile is seen for Sample 2 due to a longer etching time, responsible for a larger lateral erosion of the crests. In the inset of Figure 1b, we show a photograph of Sample 1 which highlights that optical functionalization of the nanorippled MoS_2_/silica interface is achieved over large-area and induces a strong light scattering efficiency.

The vibrational response of the MoS_2_ gratings has been investigated by micro-Raman spectroscopy. Figure 1e–g show the Raman spectra from a continuous 2D MoS_2_ film deposited on the flat silica substrate (black curve), Sample 1 (blue curve) and Sample 2 (red curve), respectively. The spectrum from each sample clearly shows the presence of the characteristic *E*_2*g*_^1^ and *A*_1*g*_ modes which denote the in-plane and out-of-plane vibrations of MoS_2_, respectively. The observed response in the Raman measurements (beam diameter ≈700 nm for a 100× objective with N.A.: 0.90) generates from an ensemble of crystalline grains of MoS_2_, up to 50 nm in size, that conformally cover the silica grating.^37^ Specifically, the frequency separation of the *E*_2*g*_^1^ to *A*_1*g*_ Raman modes for the 2D MoS_2_ film amounts to 24–25 cm^–1^, compatible with 4–5 layers and an absolute thickness of 2.8–3.5 nm (considering the thickness of a monolayer 0.7 nm). Such figures are independently in agreement with TEM characterization of the reference MoS_2_ films grown in the same conditions.^52,53^

**Figure 2:**
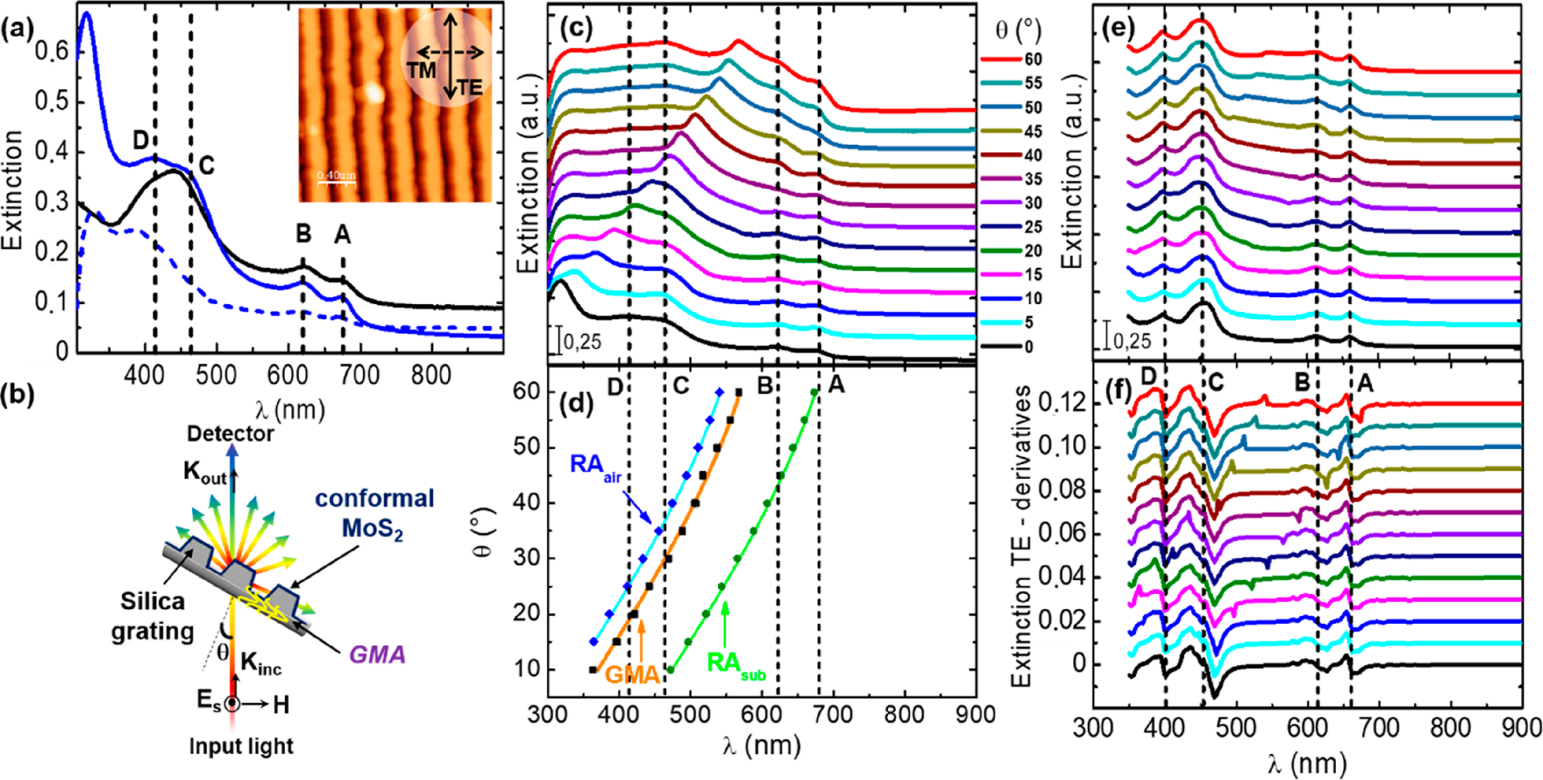
(a) Normal incidence optical extinction spectra for a reference flat MoS_2_ film (continuous black) and for Sample 1 (solid blue: TE polarization, dashed blue: TM polarization). Inset shows the direction of polarization superimposed on the AFM image of the MoS_2_ coated gratings (Sample 1). (b) Schematic of the transmission setup used for the angle-resolved optical measurements. The incident light is s-polarized and parallel to the long-axis of the MoS_2_ grating. (c) Angle resolved extinction spectra of MoS_2_ grating (Sample 1) for increasing incidence, θ, from 0° (black curve) to 60° (red curve). A relative offset of 0.2 has been introduced from one spectrum to the other for clarity purpose. (d) Comparison of the resonant wavelength for the experimentally observed diffractive anomaly (black squares, continuous orange) with RA modes captured through computations; RA_air_ (blue diamonds) and RA_sub_ (green circles). Simulated angle-resolved extinction spectra in s-TE polarization for Sample 1. (f) Numerical derivatives of the simulated extinction spectra of panel 2e, aimed at identifying the RAs of Sample 1.

The uniform periodic modulation of the 2D layer over a large area enables us to measure the optical response of these metasurfaces via NUV–VIS−NIR extinction spectroscopy by exploiting a macroscopic optical spot (diameter ≈2 mm).^54^Figure 2a shows the recorded optical extinction spectra at normal incidence from a flat 2D MoS_2_ film and from a 2D MoS_2_ grating (Sample 1) for polarization of the electric field oriented parallel (TE) and perpendicular (TM) with respect to the long-axis of the nanoscale grating (see inset in Figure 2a). Each spectrum is referenced to air, including the contribution of reflection from the silica substrate. Of relevance for the photon harvesting functionality, we evidence that the subwavelength nanotexturing of the MoS_2_/silica interface induces a substantial reduction of the Fresnel reflection losses (in the order of 50%) due to moth-eye effect.^50,55,56^ For instance, in Figure 2a one can see that above 750 nm, where absorption of MoS_2_ is negligible, extinction is determined by the reflection at the air/MoS_2_ interface and drops from about 9% for the flat MoS_2_ film to about 4% from the rippled film.

The characteristic neutral excitonic resonances in the 2D MoS_2_ are readily recognized in all spectra, where the low energy peaks labeled as A and B generate from spin–orbit splitting of the valence band at K-point of the Brillouin zone and the high energy C and D excitons are due to the band-nesting transitions at the edges of Brillouin zone.^49,57^ The polarization dependent optical response detected in the case of MoS_2_ gratings (Sample 1) clearly shows a strong dichroism with enhanced extinction over a broadband NUV–VIS spectrum for TE polarization (solid blue curve) with respect to TM polarization (dashed blue curve). In particular, for TE polarization a narrowband optical extinction maximum is detected at the wavelength λ ≈ 320 nm, giving rise to a relative enhancement of about 180% with respect to the corresponding signal of the reference flat MoS_2_ film.

The presence of an anomalous peak at λ ≈ 320 nm leading to a strong extinction enhancement invokes a detailed investigation; therefore, we performed angle resolved optical extinction measurements for Sample 1 (see Figure 2b for a schematic of the adopted setup). In detail, the MoS_2_ coated grating was illuminated by a NUV–VIS−NIR light from the substrate side in s-TE polarization tilting the sample at various angular configurations, thus allowing us to control the interaction between the incident light and the wave-vector component of MoS_2_ grating (2π/*D*). Figure 2c shows the optical extinction spectra for 0 ≤ ° θ ≤ 60°. A clear and gradual red-shift of the sharp spectral feature is seen from 320 to 565 nm as θ increases from 0° (black curve) to 60° (red curve). The dispersive nature of the peak is characteristic of photonic anomalies observed for highly ordered periodic arrays that can be classified to be either a Rayleigh Anomaly (RA) or a guided mode anomaly (GMA).^58,59^ In particular, RA refers to diffracted light propagating parallel to the grating plane whereas in the GMA regime,^58^ the diffracted wave can couple with the underlying substrate therein propagating with the wave-vector parallel to the short axis of the grating. The latter behavior is possible due to the larger value of the refractive index in the substrate with respect to the effective medium formed by the diffraction grating and the surrounding medium that allows for total internal reflection of the wave coupled to the substrate. As the GMA wave is guided through the substrate, it can be further coupled to the 2D layer leading to resonant and nonresonant interactions of the scattered light with the nanograting. The dependence of the peak wavelength (*λ*_*p*_) for the two diffractive anomalies on the illumination angle (θ) can be explained by the following equation:^58,59^
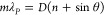
1where *m* is the order of diffraction, *D* is the grating period, and *n* is the refractive index of the medium for RA excited in the bare silica grating. In particular, in the GMA induced at the MoS_2_–silica interface, *n* corresponds to the effective refractive index of the hybrid MoS_2_–silica metastructure. As shown in Figure 2d, the photonic anomaly of Figure 2c could be fitted by using eq 1 and setting *D* = 290 nm and *m* = 1 for varying θ. A very good match is found between the experiment (black squares) and the model (orange line) when an effective refractive index *n* = 1.07 ± 0.05 is adopted for the MoS_2_ coated silica metastructure. In panel 2d the error bars are not visible because they are comparable or smaller than the symbol size. In particular the angular accuracy of the goniometer reads about 1° and the wavelength resolution at the resonant peaks amounts to 1 nm. The repeatability of the optical spectra measured in different spots, separated by macroscopic distance, is demonstrated in Supporting Information (SI) Figure S5 which helps to better highlight the high degree of lateral homogeneity in Sample 1.

These flat optic silica gratings homogeneously coated with atomically thin MoS_2_ layers demand for electromagnetic computations aiming at a detailed investigation of their optical response. To this end we use Finite Element Method (FEM) analysis assuming as model structure a single ridge morphology, resulting from the AFM line profiles of Sample 1 (Figure 1c) and Sample 2 (Figure 1d), respectively. In Figure 1h,i we show the schematic of the unit cell with the grating geometry employed for simulating Samples 1 and 2, respectively, highlighting the regions with different refractive indexes (in the sketch the thickness of the MoS_2_ layer is exaggerated for the sake of visibility). To investigate the nature and the coupling of the diffractive anomaly to 2D MoS_2_, we performed FEM computations for Sample 1 (*D* = 290 nm). To this end we use a commercial FEM solver (Comsol Multiphysics 5.4) which assumes as model structure the single ridge morphology represented in panel 1h, resulting from the AFM line profiles of Sample 1 which are evidenced in panel 1c. The simulated MoS_2_ coated grating is excited with s-TE polarized light at a specific illumination angle, θ. Figure 2e shows the calculated spectrum for each θ bringing out the characteristic neutral excitonic resonances and two different modes of the Rayleigh Anomaly that red-shift with the increase in the incident angle (the latter have been clearly identified by inspecting the numerical derivative of the simulated spectra, as detailed in the Figure 2f). We attribute the high energy mode to the scattering of incident light along the MoS_2_ grating–air interface (RA_air_), whereas the low energy mode (RA_sub_) emerges due to the scattering of incident light along the MoS_2_ grating–silica interface. These modes arise due to the different refractive indices of the media surrounding the periodically modulated MoS_2_ layer, on one side the air (*n* = 1.0) for RA_air_, and on the other side the silica (*n* = 1.46) for RA_sub_, thus allowing for a change in the magnitude of the wave-vector component of the scattered light in the two media. In particular, the RA_air_ spans through the high energy C and D excitonic resonances, while the RA_sub._ moves through the low energy A and B excitonic resonances of the 2D MoS_2_ film. Figure 2d also shows the dispersion of the resonant wavelengths for the two simulated RA modes (RA_air_–blue trace and diamonds, RA_sub_–green trace and circles) compared to the experimental data (black squares) which where fitted by using eq 1. The discrepancy between the experimental position of the photonic anomaly and the values of the RA anomalies is due to occurrence of a GMA confined in the silica substrate (thickness ≈ 700 μm).^58^ Indeed the finite thickness of the dielectric substrate can sustain a continuous spectrum of guided modes (see sketch in Figure 2b) which leak from the MoS_2_ coated nanotextured layer, provided its effective refractive index is lower compared to the silica supporting slab. In our case, the fit to the experimental data highlights an effective refractive index *n* = 1.07 ± 0.05 for the MoS_2_ coated silica metastructure. This value is intermediate between the refractive index of the substrate (1.46) and air (1.00), demonstrating that the observed modes are compatible with GMA leaking from the MoS_2_ coated nanopatterned surface.

**Figure 3:**
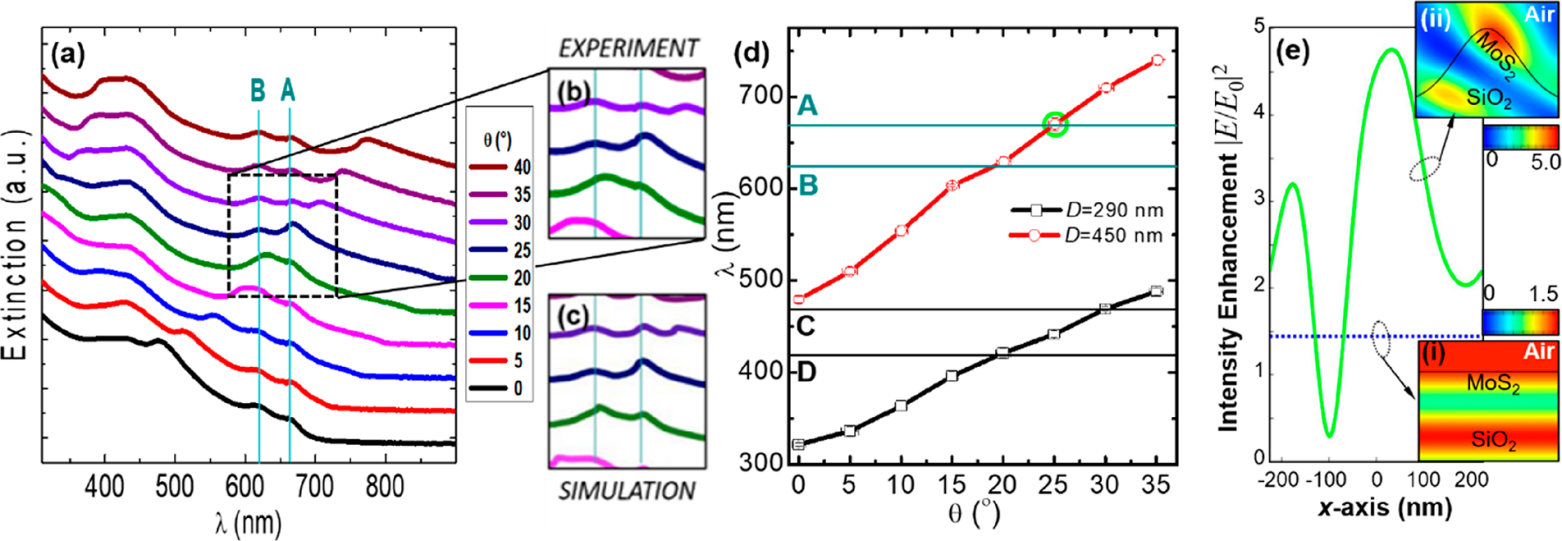
(a) Angle-resolved extinction spectra for Sample 2 (*D* = 450 nm). Experimental setup as in Figure 2b. (b,c) Focus on red-shift of the dispersion of GMA and RA_air_ through characteristic A and B excitonic resonances in 2D MoS_2_ from the experiment and simulations, respectively. (d) Dispersion of the GMA resonant wavelength plotted as a function of the illuminating angle for Samples 1 and 2. Tunability of the GMA from 300 to 750 nm is achieved by changing the grating period. (e) Simulated electric field intensity for illumination at θ = 25° at fixed light wavelength 660 nm (green circle in panel 3d) when the x-coordinate moves across the unit cell, for a flat (dotted blue) and modulated 2D MoS_2_ (continuous green), supported on a semi-infinite silica substrate. The color images in Figure 3e show the simulated magnitude of the near field for the flat film (bottom inset) and for Sample 2 (top inset).

The experimental observations in Figure 2c and the quantitative agreement with the theory of diffractive anomalies are evidence of the fact that the GMA can be tuned by varying the illumination angle. At the same time eq 1 suggests that the GMA can be independently red-shifted by increasing the grating period, thus leading to a spectral overlap of the guided-mode anomaly with low energy excitonic resonances in the active 2D layer. In Sample 2 we thus modified the grating period as *D* = 450 nm (Figure 1b,d) recording the angle resolved extinction spectra as performed previously for Sample 1. Figure 3a shows the optical extinction curves for different illumination angles leading to the red-shift of the guided mode anomaly from 475 nm at θ = 0° (black curve) to 770 nm at θ = 40° (brown curve). The larger period of the array allows the resonant mode to span over broadband wavelength range, now also including the low energy A and B excitonic resonances in MoS_2_ at illuminating angles far from grazing conditions. Figure 3b,c show the zoomed-in view of the optical extinction from the experiment and the FEM simulations assuming as model structure for Sample 2 the single ridge morphology sketched in panel 1i. Looking carefully at the dispersion of GMA and RA_air_, one can notice slight deviations in resonant wavelength between the two anomalies as they crossover low energy excitonic resonances A and B in MoS_2_. Figure 3d shows a comparison between the dispersion of GMA wavelength for Sample 1 and 2 for θ varying between 0° and 35°. The figure clearly elucidates the dependence of the GMA upon the grating period and the illuminating angle; whereas for Sample 1 (*D* = 290 nm), it is possible to tune the resonant wavelength of the diffractive anomaly from NUV to VIS across the high energy C and D excitonic resonances of MoS_2_ (open black squares), the larger period of Sample 2 (*D* = 450 nm) allows the GMA to crossover low energy A and B excitonic resonances (open red circles). Thus, a simple modification of the period of the nanopatterned template allows us to enhance interaction of incident photons with specific MoS_2_ excitonic resonances spanning from low to high energy. Figure 3e shows the simulated electric field intensity in s-TE polarization for θ = 25° at fixed illumination wavelength 660 nm for Sample 2 and for the flat MoS_2_, as a function of the distance along the unit cell. The values of angle and wavelength have been chosen in order to have the resonant excitation of GMA for Sample 2 in proximity to the A exciton in the 2D MoS_2_. The calculated intensity of the electric field shows a clear enhancement (up to 3-fold) for Sample 2 (solid green) when compared to the flat MoS_2_ film (dotted blue). The comparison between the near-field cross-section profile of the flat layer (Figure 3e bottom inset) and the patterned layer (Figure 3e top inset) permits to identify in a straightforward way the selective confinement of electromagnetic hot-spots on the illuminated MoS_2_ ridges of the patterned sample. These results clearly show the capability to tailor photon trapping and absorption in corrugated 2D MoS_2_ nanosheets, by simply reshaping the 2D material and tailoring the periodicity of the underlying grating nanopattern. This is a promising outcome for the use of 2D TMD coated gratings for amplifying excitonic absorption along with applications devoted to photocatalysis and sensing over a broad wavelength range.

**Figure 4:**
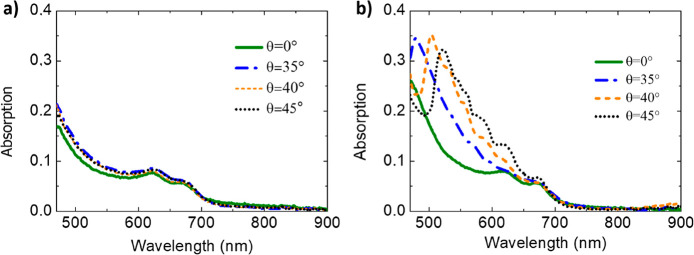
Optical absorption spectra extracted from integral transmission measurements performed under different incidence angle illumination conditions and s-TE polarization of the incident light for (a) flat MoS_2_ film and (b) MoS_2_ nanogratings corresponding to Sample 1 (periodicity *D* = 290 nm), respectively.

In order to highlight the impact of the proposed nanopatterned configuration for enhanced photon absorption in few-layer 2D MoS_2_, we performed measurements of total integrated absorbance using an integrating sphere setup,^54^ able to collect the integral optical transmission through the sample which is mounted inside the integrating sphere. To this end, the integral transmittance spectra of the rippled MoS_2_ films corresponding to Samples 1 and 2 have been detected and compared with the spectra of a reference flat MoS_2_ film. The possibility to control the incidence angle (θ) of the excitation beam on the sample has also allowed to collect a set of data that characterizes the angular dispersion of the detected absorption features in MoS_2_ nanogratings. All the spectra were detected for s-TE polarization of the excitation beam, as sketched in Figure 2b. The optical setup allows us to detect the total integrated signal (*T*) due to direct transmission, specular reflection, and diffused forward/backward scattering at any angle, giving the optical absorption from the sample as *A* = 1 – *T* (see the Methods section for details). Figure 4a,b shows the absorption spectra respectively corresponding to the flat MoS_2_ film and to the MoS_2_ nanogratings (Sample 1). The spectra have been detected under different illumination angles θ = 0° (green), θ = 35° (blue), θ = 40° (orange), and θ = 45° (black). In all the spectra referred to periodically modulated MoS_2_ (Figure 4b, Sample 1) we observe a broadband increase of optical absorption in the whole active window of MoS_2_, with respect to the case of the reference flat layer (Figure 4a). Additionally, an absorption maximum is clearly detected as θ exceeds 30° and can be tuned from about 480 to 525 nm wavelength by tailoring the light incidence angle θ from 35° to 45°. Such dispersive behavior is in agreement with the red-shift of the extinction maximum detected in Figure 2c and can be clearly attributed to the excitation of a GMA propagating at the nanopatterned silica/MoS_2_ interface. Under GMA excitation at θ = 45° in MoS_2_ nanogratings, a resonant absorption enhancement as high as 240% is detected with respect to the corresponding signal from a flat 2D layer. This effect is not merely confined to the GMA resonant wavelength but extends over a broadband VIS spectral range from 470 to 750 nm, with an averaged absorption gain which reads about 110% at θ = 45°. This enhancement is due to the interaction of the nonresonant scattered light, guided into the bulk substrate, with the 2D nanogratings.^57,58^ Similar optical behavior has been observed for Sample 2 (SI Figure S4) where we observe a narrowband maximum at 490 nm wavelength for normal incident excitation (θ = 0°, pink curve). This characteristic feature red-shifts to about 504 nm wavelength for θ = 5°, showing a dispersive behavior in agreement with that of the GMA mode observed in the extinction spectra (Figure 3a–d). In analogy to Sample 1 a broadband absorption amplification effect is detected in the whole MoS_2_ active band, confirming the interaction of nonresonant scattered light with the nanogratings.

We thus demonstrated the potential of this flat-optics approach combining both resonant light confinement and nonresonant wave guiding in the dielectric template for broadband photon-harvesting in few-layer MoS_2_. Note that, contrary to the case of MoS_2_ monolayers and bilayers, a 4/5-layer MoS_2_ nanosheet can be described in terms of a bulk permittivity. It is therefore expected that the kind of absorption enhancement retrieved in our study cannot be affected by slight (local) variations in the number of layers. However, for MoS_2_ thickness beyond 8–10 nm the onset of retardation-based regime in the nanosheets is expected in view of the very high optical density of MoS_2_. For such optically thick MoS_2_ structures, the GMA mechanism can take place also within the MoS_2_ film itself, giving rise to a further enhancement of absorption, even though on a narrow range of wavelengths. Given the large (cm^2^) area and high throughput nature of the novel approach here described, we envisage a strong impact in view of possible development of scalable 2D layered nanophotonic devices featuring broadband photon harvesting.

## 3 Conclusion

A novel photon harvesting scheme for few-layer 2D MoS_2_ nanogratings has been demonstrated which exploits large-area dielectric templates extending over cm^2^ areas. A subwavelength silica grating conformally coated with large-area 2D MoS_2_ layers promotes light coupling within the 2D material thanks to the excitation of a guided mode anomaly confined at the substrate–2D layer interface. The dispersive GMA feature enables a strong resonant absorption of photons into the MoS_2_ layers and is easily tunable over a broadband range from the NUV to the NIR by simply tailoring the illumination conditions and/or the grating periodicity. In parallel, a further broadband amplification of absorption is achieved in the VIS spectral range from 470 to 750 nm thanks to a nonresonant light scattering from the nanogratings which leads to an enhancement of integrated photon absorption up to 110% in nanorippled 2D MoS_2_ with respect to the reference flat MoS_2_. Furthermore, the moth-eye antireflection effect induced in the VIS-NIR wavelength range by the MoS_2_ coated gratings, determines a further route toward efficient light collection. The strong and broadband photon harvesting efficiency of these flat optics configurations opens the possibility to exploit this large area nanofabrication approach in a new generation of macroscopic 2D nanodevices with a strong impact in nanophotonics, photovoltaics, and quantum technologies.

## Methods

### Fabrication of MoS_2_ Gratings

The growth of few-layer conformal MoS_2_ films on silica gratings was achieved by combining Lloyd Mirror Laser Interference Lithography (LIL) technique with Reactive Ion Etching (RIE) followed by conformal deposition of MoS_2_ film. In particular, the following fabrication steps have been performed (SI Figure S1).

A. Spin coating and soft baking of a bare silica substrate, previously washed in acetone and isopropyl alcohol, with a thin layer of diluted positive photoresist (AZ MIR 701 thinned with AZ EBR solvent). We employed our customized laser interference lithography (LIL) setup in Lloyd configuration in order to impress an optical interference fringe pattern onto the polymer. The polymer was then exposed to AZ 726 MIF developer to achieve large area array of laterally separated polymer stripes. The parameters involved in LIL technique were optimized to develop a fringe pattern with desired periodicity between the stripes of the polymer resist (see SI Table S1 for parameters employed for Samples 1 and 2). B. The second step involves preparing the polymer templates for RIE. To this end, Al mask was deposited on the polymer stripe pattern at normal incidence followed by removal of polymer stripes using ultrasonication in acetone for an optimized time duration. The Al stripe covered silica substrate was then processed through RIE for developing well-ordered silica gratings with controlled surface profile (see SI Table S2 for chosen RIE parameters for Samples 1 and 2). C. For MoS_2_ synthesis, 4 nm thick MoO_3_ films were evaporated on the flat substrate and silica gratings by means of electron beam evaporation. The latter were then exposed to sulfur enriched atmosphere in a tubular furnace by using Ar gas with flow rate 0.2–0.3 L/h to transport sulfur from the source (1–2 gr., Sigma-Aldrich) to the sample. The furnace was heated to 850 °C with 5 °C/min rate and kept at this high temperature for 10 min followed by natural cooling, achieving growth of conformal MoS_2_ thin films on the silica gratings.

### Atomic Force Microscopy

The AFM imaging of MoS_2_ flat continuous film for Samples 1 and 2 has been performed by a Nanomagnetics ezAFM Instrument operating in tapping mode. The system is equipped with a high aspect ratio Si tip (radius of curvature ≈10 nm) supplied by NANOSENSORS. Typically, the images were acquired with the fast axis of scanner parallel to the wave-vector component of the MoS_2_ grating. Square imaging (area: 4 μm^2^) was performed for all samples with 512 points along vertical and horizontal scan axes.

### Micro-Raman Spectroscopy

We utilized Hitachi Xplora Plus Raman spectrometer to measure Raman Shift from the samples. The measurement was performed in backscattering condition. The system is equipped with a liquid nitrogen cooled CCD, 100× objective lens (N.A. = 0.90) and several gratings. The excitation wavelength was 532 nm. A 2400 lines/mm grating was used which gives a spectral resolution of 0.69 cm^–1^. The input laser power was fixed to 1 mW so as prevent any damage to the sample. Ten different spots were measured on the surface so as to determine the uniformity of the sample. The acquisition time for each signal was fixed to 20 s with 10 accumulations.

### Far-Field Optical Spectroscopy

The optical response of MoS_2_ gratings was studied by ex-situ extinction spectroscopy and integrating sphere. We utilized a spectrometer from Ocean Optics (HR4000) operating in the wavelength range 300−1100 nm. The incident light was obtained through a Halogen and a deuterium source (DH-2000-BAL, Mikropak) that was coupled to an optical fiber with core diameter 600 μm. The light after passing through the sample is collected by another optical fiber coupled to the PC controlled detector. Raw transmittance data, normalized to an uncoated flat silica substrate is shown for reference 2D MoS_2_ flat film, Sample 1 in SI Figure S2a and Sample 2 in SI Figure S2b.

### Angle Resolved Extinction

A collimated beam (≈ diameter 2 mm) generated through a Halogen and deuterium lamp (DH-2000-BAL, Mikropak) illuminates the sample through an optical fiber (core diameter 600 μm) from the substrate side. The chosen polarization configuration is S-TE where the electric field vector vibrates parallel to the long axes of the MoS_2_ coated silica grating. The sample stage fixed to a goniometer is then rotated from θ = 0° to 60° (Sample 1) and θ = 0° to 45° (Sample 2) to initiate the interaction of nanorippled MoS_2_ ultrathin films with light at various angles of incidence. The accuracy of our goniometer is ±1° and we increase the angular position at 5° steps. The detector (Ocean Optics HR4000 operating in the wavelength range 300–1100 nm) coupled to the PC through an optical fiber (core diameter 600 μm) remains fixed to collect transmission (*T*) signal parallel to the normal of the sample and the incident beam of light. The optical extinction is then calculated as 1 – *T*.

### Absolute Absorption from Integrating Sphere

To quantify absorption from our samples, we used a NKT Photonics broadband white laser (SuperK COMPACT) as an illumination source coupled to an integrating sphere through a polarizer. The output from the sphere was coupled to the PC controlled HR4000, Ocean Optics spectrometer through an optical fiber (core diameter 600 μm). We measured the optical absorption in the following manner. Step 1: a constant dark spectrum was acquired with light source off so as to read the detector noise level. Step 2: integrated signal was recorded in indirect illumination (*T*_indirect_, sample kept within the sphere and away from the incident light to capture optical background) followed by the recording of the signal in direct illumination (*T*_direct_, sample mounted in the line of sight of the incident light). The normalized integrated signal (*T*) is then given by (*T*_direct_-dark)/(*T*_indirect_-dark). The total absorption is evaluated as *A* = (1 – *T*).

### Numerical Simulations

For the numerical simulations of the MoS_2_ grating, we employed a commercial tool (Comsol Multiphysics 5.4) implementing the finite-element method in the frequency domain. A two-dimensional configuration is considered with lateral Bloch-Floquet boundary conditions defining the grating period (Sample 1: 290 nm, Sample 2: 450 nm) along the horizontal direction. To mimick the actual thickness of the 4/5 layers of MoS_2_ in the fabricated sample, the unit cell comprises a 4 nm thick MoS_2_ film supported on a homogeneous lossless and nondispersive silica substrate. The air cover and the silica substrate are modeled as semi-infinite media by imposing port boundary conditions on top and bottom edges of the unit cell. For MoS_2_ dielectric function, we assumed the same description reported in our previous work (ref (48)), that is, a complex tensor with two nondegenerate (in-plane and out-of-plane) spectral components. The MoS_2_ domain was discretized with triangular mesh elements with maximum size of 1 nm, whereas for the substrate and cover domains, the maximum size of mesh elements was fixed to 30 and 50 nm, respectively. For field intensity enhancement evaluation, this is provided by taking the ratio, modulus square, between the total electric field E calculated along the median contour of the MoS_2_ nanosheet and the electric field E_0_ of the plane wave impinging on the structure. Note that the local absorption in a lossy dielectric being proportional to the local intensity of the electric field, the field intensity enhancement in the MoS_2_ domain correlates with the absorption of the structure.

## Supporting Information Available

Fabrication of MoS_2_ grating, optical transmittance raw spectra for Samples 1 and 2, large area pictures of Sample 2 and flat reference, integrated optical absorption spectra of Sample 2, comparison of extinction spectra acquired at different locations of the sample to highlight sample uniformity on a macroscopic scale (PDF)

## References

[ref1] Fabbri F.; Rotunno E.; Cinquanta E.; Campi D.; Bonnini E.; Kaplan D.; Lazzarini L.; Bernasconi M.; Ferrari C.; Longo M.; Nicotra G.; Molle A.; Swaminathan V.; Salviati G. Novel Near-Infrared Emission from Crystal Defects in MoS_2_ Multilayer Flakes. Nat. Commun. 2016, 7 (1), 13044 10.1038/ncomms13044. 27698425 PMC5059461

[ref2] Liu H.; Li Y.; You Y. S.; Ghimire S.; Heinz T. F.; Reis D. A. High-Harmonic Generation from an Atomically Thin Semiconductor. Nat. Phys. 2017, 13 (3), 262–265. 10.1038/nphys3946.

[ref3] Choi W.; Choudhary N.; Han G. H.; Park J.; Akinwande D.; Lee Y. H. Recent Development of Two-Dimensional Transition Metal Dichalcogenides and Their Applications. Mater. Today 2017, 20 (3), 116–130. 10.1016/j.mattod.2016.10.002.

[ref4] Chhowalla M.; Shin H. S.; Eda G.; Li L.-J.; Loh K. P.; Zhang H. The Chemistry of Two-Dimensional Layered Transition Metal Dichalcogenide Nanosheets. Nat. Chem. 2013, 5 (4), 263–275. 10.1038/nchem.1589. 23511414

[ref5] Arnold A. J.; Razavieh A.; Nasr J. R.; Schulman D. S.; Eichfeld C. M.; Das S. Mimicking Neurotransmitter Release in Chemical Synapses *via* Hysteresis Engineering in MoS _2_ Transistors. ACS Nano 2017, 11 (3), 3110–3118. 10.1021/acsnano.7b00113. 28260370

[ref6] Purcell-Milton F.; McKenna R.; Brennan L. J.; Cullen C. P.; Guillemeney L.; Tepliakov N. V.; Baimuratov A. S.; Rukhlenko I. D.; Perova T. S.; Duesberg G. S.; Baranov A. V.; Fedorov A. V.; Gun’ko Y. K. Induction of Chirality in Two-Dimensional Nanomaterials: Chiral 2D MoS _2_ Nanostructures. ACS Nano 2018, 12 (2), 954–964. 10.1021/acsnano.7b06691. 29338193

[ref7] Zhou X.; Hu X.; Yu J.; Liu S.; Shu Z.; Zhang Q.; Li H.; Ma Y.; Xu H.; Zhai T. 2D Layered Material-Based van Der Waals Heterostructures for Optoelectronics. Adv. Funct. Mater. 2018, 28 (14), 1706587 10.1002/adfm.201706587.

[ref8] Yu Y.; Hu S.; Su L.; Huang L.; Liu Y.; Jin Z.; Purezky A. A.; Geohegan D. B.; Kim K. W.; Zhang Y.; Cao L. Equally Efficient Interlayer Exciton Relaxation and Improved Absorption in Epitaxial and Nonepitaxial MoS _2_ /WS _2_ Heterostructures. Nano Lett. 2015, 15 (1), 486–491. 10.1021/nl5038177. 25469768

[ref9] Deng D.; Novoselov K. S.; Fu Q.; Zheng N.; Tian Z.; Bao X. Catalysis with Two-Dimensional Materials and Their Heterostructures. Nat. Nanotechnol. 2016, 11 (3), 218–230. 10.1038/nnano.2015.340. 26936816

[ref10] Huo N.; Konstantatos G. Recent Progress and Future Prospects of 2D-Based Photodetectors. Adv. Mater. 2018, 30 (51), 1801164 10.1002/adma.201801164. 30066409

[ref11] Toth M.; Aharonovich I. Single Photon Sources in Atomically Thin Materials. Annu. Rev. Phys. Chem. 2019, 70 (1), 123–142. 10.1146/annurev-physchem-042018-052628. 30735459

[ref12] Mahyavanshi R. D.; Desai P.; Ranade A.; Tanemura M.; Kalita G. Observing Charge Transfer Interaction in CuI and MoS _2_ Heterojunction for Photoresponsive Device Application. ACS Applied Electronic Materials 2019, 1 (3), 302–310. 10.1021/acsaelm.8b00069.

[ref13] Mak K. F.; Shan J. Photonics and Optoelectronics of 2D Semiconductor Transition Metal Dichalcogenides. Nat. Photonics 2016, 10 (4), 216–226. 10.1038/nphoton.2015.282.

[ref14] Wang Q. H.; Kalantar-Zadeh K.; Kis A.; Coleman J. N.; Strano M. S. Electronics and Optoelectronics of Two-Dimensional Transition Metal Dichalcogenides. Nat. Nanotechnol. 2012, 7 (11), 699–712. 10.1038/nnano.2012.193. 23132225

[ref15] Matković A.; Petritz A.; Schider G.; Krammer M.; Kratzer M.; Karner-Petritz E.; Fian A.; Gold H.; Gärtner M.; Terfort A.; Teichert C.; Zojer E.; Zojer K.; Stadlober B. 2D Semiconductors: Interfacial Band Engineering of MoS _2_ /Gold Interfaces Using Pyrimidine-Containing Self-Assembled Monolayers: Toward Contact-Resistance-Free Bottom-Contacts (Adv. Electron. Mater. 5/2020). Advanced Electronic Materials 2020, 6 (5), 2070026 10.1002/aelm.202070026.

[ref16] Schaibley J. R.; Yu H.; Clark G.; Rivera P.; Ross J. S.; Seyler K. L.; Yao W.; Xu X. Valleytronics in 2D Materials. Nat. Rev. Mater. 2016, 1 (11), 16055 10.1038/natrevmats.2016.55.

[ref17] Cortés N.; Ávalos-Ovando O.; Rosales L.; Orellana P. A.; Ulloa S. E. Tunable Spin-Polarized Edge Currents in Proximitized Transition Metal Dichalcogenides. Phys. Rev. Lett. 2019, 122 (8), 086401 10.1103/PhysRevLett.122.086401. 30932605

[ref18] Voiry D.; Yang J.; Chhowalla M. Recent Strategies for Improving the Catalytic Activity of 2D TMD Nanosheets Toward the Hydrogen Evolution Reaction. Adv. Mater. 2016, 28 (29), 6197–6206. 10.1002/adma.201505597. 26867809

[ref19] Krasnok A.; Lepeshov S.; Alú A. Nanophotonics with 2D Transition Metal Dichalcogenides [Invited]. Opt. Express 2018, 26 (12), 15972 10.1364/OE.26.015972. 30114850

[ref20] Luo Z.; Wu D.; Xu B.; Xu H.; Cai Z.; Peng J.; Weng J.; Xu S.; Zhu C.; Wang F.; Sun Z.; Zhang H. Two-Dimensional Material-Based Saturable Absorbers: Towards Compact Visible-Wavelength All-Fiber Pulsed Lasers. Nanoscale 2016, 8 (2), 1066–1072. 10.1039/C5NR06981E. 26658877

[ref21] Wang G.; Baker-Murray A. A.; Blau W. J. Saturable Absorption in 2D Nanomaterials and Related Photonic Devices. Laser Photonics Rev. 2019, 13 (7), 1800282 10.1002/lpor.201800282.

[ref22] Zhang S.; Dong N.; McEvoy N.; O’Brien M.; Winters S.; Berner N. C.; Yim C.; Li Y.; Zhang X.; Chen Z.; Zhang L.; Duesberg G. S.; Wang J. Direct Observation of Degenerate Two-Photon Absorption and Its Saturation in WS _2_ and MoS _2_ Monolayer and Few-Layer Films. ACS Nano 2015, 9 (7), 7142–7150. 10.1021/acsnano.5b03480. 26135798

[ref23] Dai X.; Zhang X.; Kislyakov I. M.; Wang L.; Huang J.; Zhang S.; Dong N.; Wang J. Enhanced Two-Photon Absorption and Two-Photon Luminescence in Monolayer MoS _2_ and WS _2_ by Defect Repairing. Opt. Express 2019, 27 (10), 13744 10.1364/OE.27.013744. 31163833

[ref24] Lin K.-I.; Ho Y.-H.; Liu S.-B.; Ciou J.-J.; Huang B.-T.; Chen C.; Chang H.-C.; Tu C.-L.; Chen C.-H. Atom-Dependent Edge-Enhanced Second-Harmonic Generation on MoS _2_ Monolayers. Nano Lett. 2018, 18 (2), 793–797. 10.1021/acs.nanolett.7b04006. 29327927

[ref25] Lin X.; Liu Y.; Wang K.; Wei C.; Zhang W.; Yan Y.; Li Y. J.; Yao J.; Zhao Y. S. Two-Dimensional Pyramid-like WS _2_ Layered Structures for Highly Efficient Edge Second-Harmonic Generation. ACS Nano 2018, 12 (1), 689–696. 10.1021/acsnano.7b07823. 29294288

[ref26] Zhang J.; Ye M.; Bhandari S.; Muqri A. K. M.; Long F.; Bigham S.; Yap Y. K.; Suh J. Y. Enhanced Second and Third Harmonic Generations of Vertical and Planar Spiral MoS _2_ Nanosheets. Nanotechnology 2017, 28 (29), 295301 10.1088/1361-6528/aa7825. 28594335

[ref27] Autere A.; Jussila H.; Marini A.; Saavedra J. R. M.; Dai Y.; Säynätjoki A.; Karvonen L.; Yang H.; Amirsolaimani B.; Norwood R. A.; Peyghambarian N.; Lipsanen H.; Kieu K.; de Abajo F. J. G.; Sun Z. Optical Harmonic Generation in Monolayer Group-VI Transition Metal Dichalcogenides. Phys. Rev. B: Condens. Matter Mater. Phys. 2018, 98 (11), 115426 10.1103/PhysRevB.98.115426.

[ref28] Bernardi M.; Palummo M.; Grossman J. C. Extraordinary Sunlight Absorption and One Nanometer Thick Photovoltaics Using Two-Dimensional Monolayer Materials. Nano Lett. 2013, 13 (8), 3664–3670. 10.1021/nl401544y. 23750910

[ref29] Giordano M. C.; de Mongeot F. B. Anisotropic Nanoscale Wrinkling in Solid-State Substrates. Adv. Mater. 2018, 30 (30), 1801840 10.1002/adma.201801840. 29882306

[ref30] Barelli M.; Mazzanti A.; Giordano M. C.; Della Valle G.; Buatier de Mongeot F. Color Routing via Cross-Polarized Detuned Plasmonic Nanoantennas in Large-Area Metasurfaces. Nano Lett. 2020, 20 (6), 4121–4128. 10.1021/acs.nanolett.9b05276. 32401524 PMC7735747

[ref31] Gisbert Quilis N.; Lequeux M.; Venugopalan P.; Khan I.; Knoll W.; Boujday S.; Lamy de la Chapelle M.; Dostalek J. Tunable Laser Interference Lithography Preparation of Plasmonic Nanoparticle Arrays Tailored for SERS. Nanoscale 2018, 10 (21), 10268–10276. 10.1039/C7NR08905H. 29790495

[ref32] Fattal D.; Li J.; Peng Z.; Fiorentino M.; Beausoleil R. G. Flat Dielectric Grating Reflectors with Focusing Abilities. Nat. Photonics 2010, 4 (7), 466–470. 10.1038/nphoton.2010.116.

[ref33] Khorasaninejad M.; Capasso F. Broadband Multifunctional Efficient Meta-Gratings Based on Dielectric Waveguide Phase Shifters. Nano Lett. 2015, 15 (10), 6709–6715. 10.1021/acs.nanolett.5b02524. 26372331

[ref34] Chowdhury D.; Giordano M. C.; Manzato G.; Chittofrati R.; Mennucci C.; Buatier de Mongeot F. Large-Area Microfluidic Sensors Based on Flat-Optics Au Nanostripe Metasurfaces. J. Phys. Chem. C 2020, 124 (31), 17183–17190. 10.1021/acs.jpcc.0c03023.

[ref35] Chang-Hasnain C. J.; Yang W. High-Contrast Gratings for Integrated Optoelectronics. Adv. Opt. Photonics 2012, 4 (3), 379 10.1364/AOP.4.000379.

[ref36] Castellanos-Gomez A.; Roldán R.; Cappelluti E.; Buscema M.; Guinea F.; van der Zant H. S. J.; Steele G. A. Local Strain Engineering in Atomically Thin MoS _2_. Nano Lett. 2013, 13 (11), 5361–5366. 10.1021/nl402875m. 24083520

[ref37] Martella C.; Mennucci C.; Cinquanta E.; Lamperti A.; Cappelluti E.; Buatier de Mongeot F.; Molle A. Anisotropic MoS _2_ Nanosheets Grown on Self-Organized Nanopatterned Substrates. Adv. Mater. 2017, 29 (19), 1605785 10.1002/adma.201605785. 28294440

[ref38] Yang J.; Wang Z.; Wang F.; Xu R.; Tao J.; Zhang S.; Qin Q.; Luther-Davies B.; Jagadish C.; Yu Z.; Lu Y. Atomically Thin Optical Lenses and Gratings. Light Sci. Appl. 2016, 5 (3), e16046-e16046. 30167150 10.1038/lsa.2016.46 PMC6059897

[ref39] Wu J.; Zhao H.; Li Y.; Ohlberg D.; Shi W.; Wu W.; Wang H.; Tan P. Monolayer Molybdenum Disulfide Nanoribbons with High Optical Anisotropy. Adv. Opt. Mater. 2016, 4 (5), 756–762. 10.1002/adom.201500707.

[ref40] Giordano M. C.; Foti A.; Messina E.; Gucciardi P. G.; Comoretto D.; Buatier de Mongeot F. SERS Amplification from Self-Organized Arrays of Plasmonic Nanocrescents. ACS Appl. Mater. Interfaces 2016, 8 (10), 6629–6638. 10.1021/acsami.5b11843. 26824254

[ref41] Quaranta G.; Basset G.; Martin O. J. F.; Gallinet B. Recent Advances in Resonant Waveguide Gratings. Laser & Photonics Reviews 2018, 12 (9), 1800017 10.1002/lpor.201800017.

[ref42] Zhou W.; Wu Y.; Yu M.; Hao P.; Liu G.; Li K. Extraordinary Optical Absorption Based on Guided-Mode Resonance. Opt. Lett. 2013, 38 (24), 5393 10.1364/OL.38.005393. 24322266

[ref43] Byelobrov V. O.; Zinenko T. L.; Kobayashi K.; Nosich A. I. Periodicity Matters: Grating or Lattice Resonances in the Scattering by Sparse Arrays of Subwavelength Strips and Wires. IEEE Antennas Propag. Mag. 2015, 57 (6), 34–45. 10.1109/MAP.2015.2480083.

[ref44] Dumcenco D.; Ovchinnikov D.; Marinov K.; Lazić P.; Gibertini M.; Marzari N.; Sanchez O. L.; Kung Y.-C.; Krasnozhon D.; Chen M.-W.; Bertolazzi S.; Gillet P.; Fontcuberta i Morral A.; Radenovic A.; Kis A. Large-Area Epitaxial Monolayer MoS _2_. ACS Nano 2015, 9 (4), 4611–4620. 10.1021/acsnano.5b01281. 25843548 PMC4415455

[ref45] Chiappe D.; Asselberghs I.; Sutar S.; Iacovo S.; Afanas’ev V.; Stesmans A.; Balaji Y.; Peters L.; Heyne M.; Mannarino M.; Vandervorst W.; Sayan S.; Huyghebaert C.; Caymax M.; Heyns M.; De Gendt S.; Radu I.; Thean A. Controlled Sulfurization Process for the Synthesis of Large Area MoS _2_ Films and MoS _2_ /WS _2_ Heterostructures. Adv. Mater. Interfaces 2016, 3 (4), 1500635 10.1002/admi.201500635.

[ref46] Hung Y.-H.; Lu A.-Y.; Chang Y.-H.; Huang J.-K.; Chang J.-K.; Li L.-J.; Su C.-Y. Scalable Patterning of MoS _2_ Nanoribbons by Micromolding in Capillaries. ACS Appl. Mater. Interfaces 2016, 8 (32), 20993–21001. 10.1021/acsami.6b05827. 27462874

[ref47] Qi R.; Wang S.; Wang M.; Liu W.; Yan Z.; Bi X.; Huang Q. Towards Well-Defined MoS _2_ Nanoribbons on a Large Scale. Chem. Commun. 2017, 53 (70), 9757–9760. 10.1039/C7CC04647B. 28812754

[ref48] Martella C.; Ortolani L.; Cianci E.; Lamperti A.; Morandi V.; Molle A. Large-Area Patterning of Substrate-Conformal MoS2 Nano-Trenches. Nano Res. 2019, 12 (8), 1851–1854. 10.1007/s12274-019-2446-0.

[ref49] Camellini A.; Mennucci C.; Cinquanta E.; Martella C.; Mazzanti A.; Lamperti A.; Molle A.; de Mongeot F. B.; Della Valle G.; Zavelani-Rossi M. Ultrafast Anisotropic Exciton Dynamics in Nanopatterned MoS _2_ Sheets. ACS Photonics 2018, 5 (8), 3363–3371. 10.1021/acsphotonics.8b00621.

[ref50] Kuo W.-K.; Hsu J.-J.; Nien C.-K.; Yu H. H. Moth-Eye-Inspired Biophotonic Surfaces with Antireflective and Hydrophobic Characteristics. ACS Appl. Mater. Interfaces 2016, 8 (46), 32021–32030. 10.1021/acsami.6b10960. 27787981

[ref51] Martella C.; Mennucci C.; Lamperti A.; Cappelluti E.; de Mongeot F. B.; Molle A. Designer Shape Anisotropy on Transition-Metal-Dichalcogenide Nanosheets. Adv. Mater. 2018, 30 (9), 1705615 10.1002/adma.201705615. 29315869

[ref52] Vangelista S.; Cinquanta E.; Martella C.; Alia M.; Longo M.; Lamperti A.; Mantovan R.; Basset F. B.; Pezzoli F.; Molle A. Towards a Uniform and Large-Scale Deposition of MoS _2_ Nanosheets via Sulfurization of Ultra-Thin Mo-Based Solid Films. Nanotechnology 2016, 27 (17), 175703 10.1088/0957-4484/27/17/175703. 26984949

[ref53] Martella C.; Melloni P.; Cinquanta E.; Cianci E.; Alia M.; Longo M.; Lamperti A.; Vangelista S.; Fanciulli M.; Molle A. Engineering the Growth of MoS _2_ via Atomic Layer Deposition of Molybdenum Oxide Film Precursor. Advanced Electronic Materials 2016, 2 (10), 1600330 10.1002/aelm.201600330.

[ref54] Mennucci C.; Muhammad M. H.; Hameed M. F. O.; Mohamed S. A.; Abdelkhalik M. S.; Obayya S. S. A.; Buatier de Mongeot F. Broadband Light Trapping in Nanotextured Thin Film Photovoltaic Devices. Appl. Surf. Sci. 2018, 446, 74–82. 10.1016/j.apsusc.2018.02.186.

[ref55] Ji S.; Song K.; Nguyen T. B.; Kim N.; Lim H. Optimal Moth Eye Nanostructure Array on Transparent Glass Towards Broadband Antireflection. ACS Appl. Mater. Interfaces 2013, 5 (21), 10731–10737. 10.1021/am402881x. 24116953

[ref56] Wilson S. J.; Hutley M. C. The Optical Properties of “Moth Eye” Antireflection Surfaces. Opt. Acta 1982, 29 (7), 993–1009. 10.1080/713820946.

[ref57] Mueller T.; Malic E. Exciton Physics and Device Application of Two-Dimensional Transition Metal Dichalcogenide Semiconductors. npj 2D Mater. Appl. 2018, 2 (1), 29 10.1038/s41699-018-0074-2.

[ref58] Wang S. S.; Moharam M. G.; Magnusson R.; Bagby J. S. Guided-Mode Resonances in Planar Dielectric-Layer Diffraction Gratings. J. Opt. Soc. Am. A 1990, 7 (8), 1470 10.1364/JOSAA.7.001470.

[ref59] Mazulquim D. B.; Lee K. J.; Yoon J. W.; Muniz L. V.; Borges B.-H. V.; Neto L. G.; Magnusson R. Efficient Band-Pass Color Filters Enabled by Resonant Modes and Plasmons near the Rayleigh Anomaly. Opt. Express 2014, 22 (25), 30843 10.1364/OE.22.030843. 25607033

