# Supplementary material for: Broadband and Tunable Light Harvesting in Nanorippled MoS2 Ultrathin Films
Source: ACS Appl Mater Interfaces. 2021 Mar 9;13(11):13508–16. doi: 10.1021/acsami.0c20387 (PMC8041252; doi:10.1021/acsami.0c20387)
Supplement: Supplementary file 1 — am0c20387_si_001.pdf [file am0c20387_si_001.pdf]

SUPPORTING INFORMATION

Broadband and Tunable Light Harvesting in Nanorippled MoS_2 Ultra-thin Films

Mukul Bhatnagar,[†,⊥] Matteo Gardella,[†,⊥] Maria Caterina Giordano,[†] Debasree Chowdhury,[†] Carlo Mennucci,[†] Andrea Mazzanti,[‡] Giuseppe Della Valle,*[,‡] Christian Martella,[§] Pinakapani Tummala,[§] Alessio Lamperti,[§] Alessandro Molle,[§] Francesco Buatier de Mongeot*[, †]

[†] Dipartimento di Fisica, Università di Genova, Via Dodecaneso 33, 16146 Genova, Italy.

[‡] Dipartimento di Fisica and IFN-CNR, Politecnico di Milano, Piazza Leonardo da Vinci, 32 - 20133 Milano, Italy.

[§] CNR-IMM Unit of Agrate Brianza, via C. Olivetti 2, Agrate Brianza, I-20864, Italy.

* Corresponding authors: buatier@fisica.unige.it , giuseppe.dellavalle@polimi.it

[⊥] These authors contributed equally

Fabrication of MoS_2 gratings

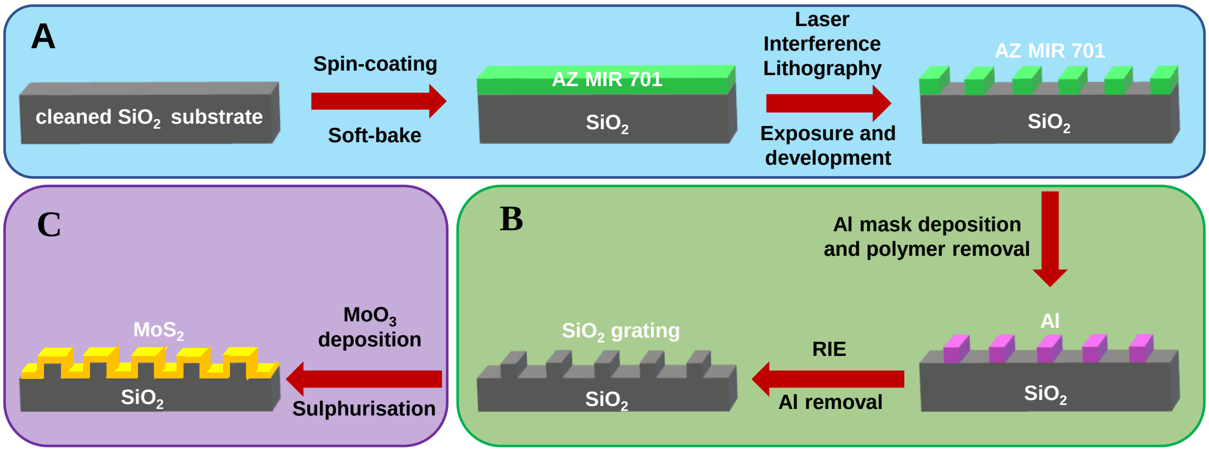

Figure S1. Schematics of fabrication of MoS_2 coated silica grating. Coloured areas recall the described steps. **A)** Spin coating and soft baking of AZMR 701 polymer resist on a cleaned silica substrate. Used laser interference lithography (LIL) to develop fringe pattern on the polymer surface followed by development to generate disconnected polymer stripes with desired period. **(B)** Al mask deposition followed by polymer removal and reactive ion etching (RIE) processing of the Al stripes leading to the formation of well-ordered silica grating. **(C)** Conformal coating of MoS_2 by electron beam deposition of MoO_3 on silica grating followed by sulphurization at 850 ^{o}C.

Table S1. LIL parameters employed for the nanofabrication of sample 1 and sample 2, respectively.

Sample	AZ MIR 701 : AZ EBR	Dilution time (min)	Laser Exposure time (sec)	Laser Power (mW)	θ w.r.t surface normal (degree)	Dose (mJ/cm^2)	Development time (sec)
1	1:2	15	25	0.497	45	20	20
2	1:2	15	35	0.414	25	23	25

Table S2. Parameters employed for Reactive Ion Etching of sample 1 and sample 2, respectively.

Sample	Aluminium thickness (nm)	Gas reactant	Power (W)	Pressure (mtorr)	RIE time (min)
1	34	Tetrafluoromethane (CF4)	100	30	6
2	54	Tetrafluoromethane (CF4)	100	30	11

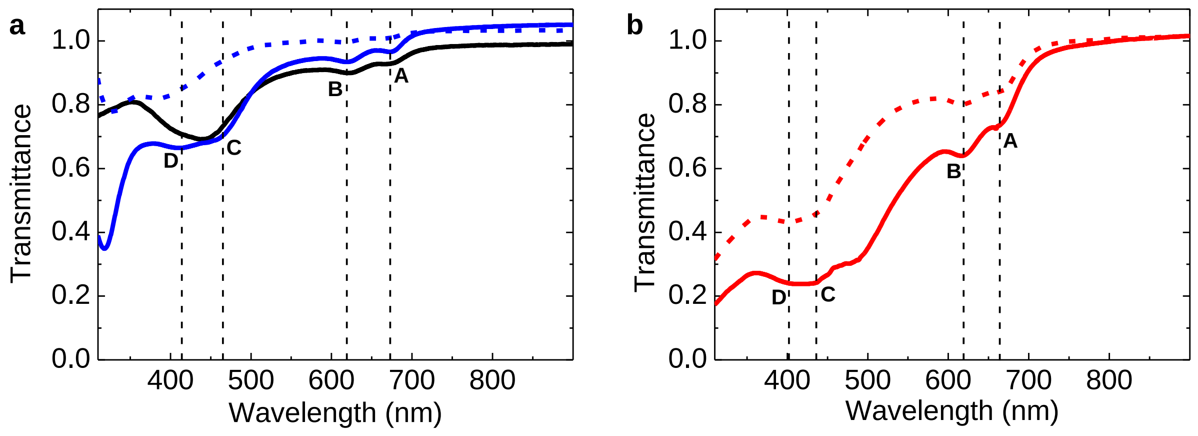

Figure S2. (a) Raw optical transmittance from a 4 nm thick 2D MoS_2 flat continuous film (black) and sample 1 (TE: continuous blue, TM: dashed blue). **(b)** Sample 2 (TE: solid red, TM: dashed red). The characteristic A,B,C,D excitonic resonances have been marked for all samples.

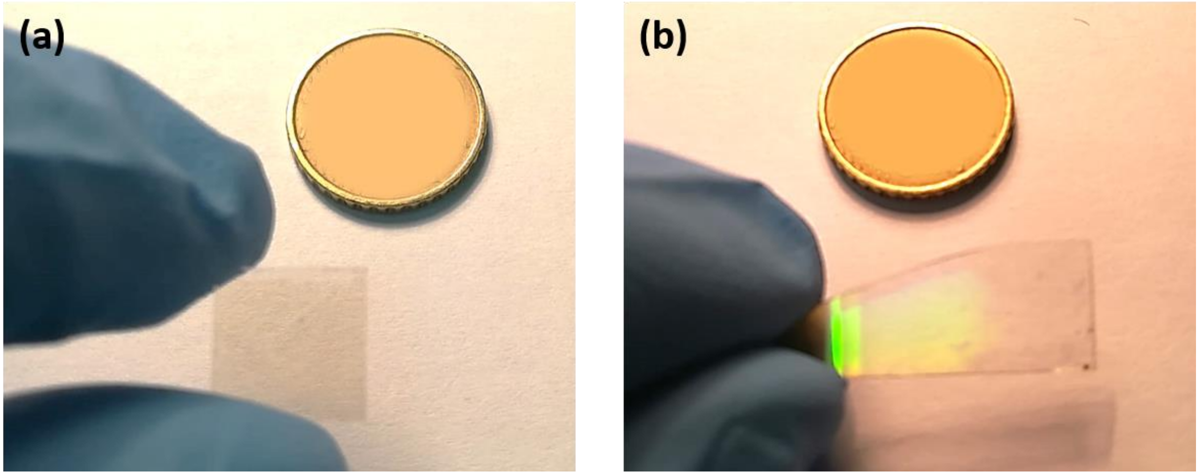

Figure S3. **(a,b)** Pictures of the large-area few-layer MoS_2 samples, respectively referred to the flat reference film and to a rippled few-layer MoS_2 nanogratings (sample 2).

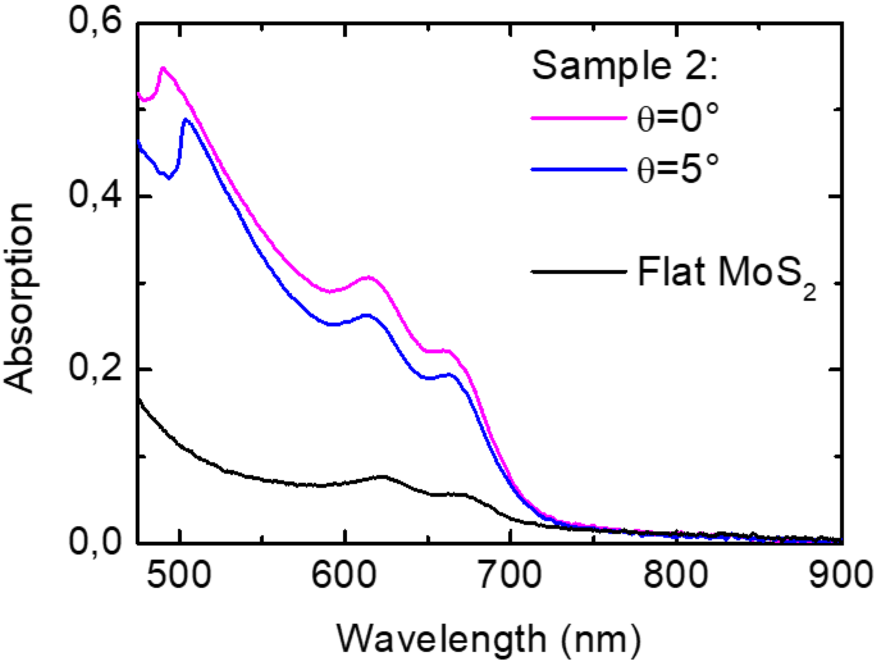

Figure S4. Optical absorption spectra extracted from integral transmission measurements performed for s-TE polarization of the incident light on a reference flat MoS_2 film (black line), and MoS_2 nanogratings corresponding to sample 2 (periodicity D = 450 nm), detected at θ=0° and 5° (pink- and blue line, respectively).

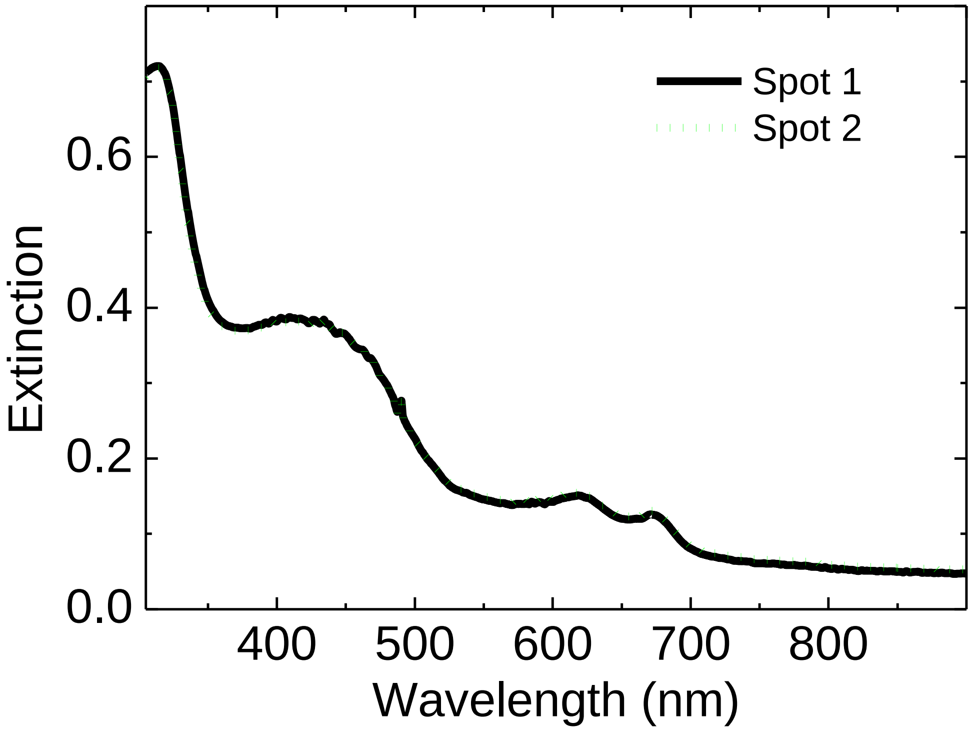

Figure S5. Optical spectra of few-layer MoS_2 detected on different positions on the same sample after a macroscopic shift. The spectral overlap of the excitonic features shows the repeatability of the optical measurements and the macroscopic homogeneity of the sample.
